# Genome-scale transcriptional analyses of first-generation interspecific sunflower hybrids reveals broad regulatory compatibility

**DOI:** 10.1186/1471-2164-14-342

**Published:** 2013-05-23

**Authors:** Heather C Rowe, Loren H Rieseberg

**Affiliations:** 1 Botany Department, University of British Columbia, Vancouver, BC, V6T 1Z4, Canada

**Keywords:** Hybridization, Helianthus, Introgression, Gene flow, Allelic bias, Speciation

## Abstract

**Background:**

Interspecific hybridization creates individuals harboring diverged genomes. The interaction of these genomes can generate successful evolutionary novelty or disadvantageous genomic conflict. Annual sunflowers *Helianthus annuus* and *H. petiolaris* have a rich history of hybridization in natural populations. Although first-generation hybrids generally have low fertility, hybrid swarms that include later generation and fully fertile backcross plants have been identified, as well as at least three independently-originated stable hybrid taxa. We examine patterns of transcript accumulation in the earliest stages of hybridization of these species via analyses of transcriptome sequences from laboratory-derived F1 offspring of an inbred *H. annuus* cultivar and a wild *H. petiolaris* accession.

**Results:**

While nearly 14% of the reference transcriptome showed significant accumulation differences between parental accessions, total F1 transcript levels showed little evidence of dominance, as midparent transcript levels were highly predictive of transcript accumulation in F1 plants. Allelic bias in F1 transcript accumulation was detected in 20% of transcripts containing sufficient polymorphism to distinguish parental alleles; however the magnitude of these biases were generally smaller than differences among parental accessions.

**Conclusions:**

While analyses of allelic bias suggest that *cis* regulatory differences between *H. annuus* and *H. petiolaris* are common, their effect on transcript levels may be more subtle than trans-acting regulatory differences. Overall, these analyses found little evidence of regulatory incompatibility or dominance interactions between parental genomes within F1 hybrid individuals, although it is unclear whether this is a legacy or an enabler of introgression between species.

## Background

For organisms that reproduce sexually, biological fitness requires the successful interaction of maternal and paternal genomes within the new individual. While these interactions may take place at various points along the path from DNA to external phenotype, analyses of transcript accumulation currently provide the strongest technology to detect these interactions on a genome-wide scale. Changes in transcript levels are hypothesized to enable response to selective forces in novel environments [1-3]. Alteration of single components of regulatory machinery may have dramatic effects on transcript profiles [4]. We therefore expect that bringing together two sets of regulatory machinery that have been separated for millions of years may lead to novel patterns of transcription that contribute to novel phenotypes in interspecific hybrids.

In plants, the effect of inter-species hybridization on transcript levels has been most extensively studied in allopolyploids, where hybridization occurs in conjunction with genome doubling. Comparison of allopolyploids with autopolyploids in several systems has provided evidence that hybridization has more dramatic effects on transcript phenotypes than increased ploidy [5-7]. In some cases, polyploidization following hybridization has been proposed as a mechanism of moderating novel transcript phenotypes generated by regulatory divergence between parental genomes [8]. Extreme gene expression changes following hybridization, or “transcriptional shock”, have been described in early-generation allopolyploid hybrids of *Arabidopsis*, wheat, and cotton, as well as diploid *Senecio* spp. hybrids [9-14]. While in later-generation hybrids and back-crosses, changes in gene expression may be caused by genome rearrangement, segregation of parental alleles, or environmentally-mediated selection on accumulated mutation, transcription in first generation (F1) hybrids will be controlled by interaction between parental genomes mediated by transcriptional machinery. Non-additive F1 transcriptional phenotypes may be caused by differences between parental species at the transcribed locus (*cis* effects) or differences in *trans*-acting regulatory factors. In hybrids, parental genomes are exposed to a common pool of trans-acting factors, and analyses of allelic bias, or differential parental genome contributions to accumulated transcript, can provide insight into the relative contributions of *cis* and *trans* effects to inter-specific gene expression differences [15,16].

The sunflower genus *Helianthus* is native to North America and contains 49 species of annual or perennial herbs. The annual sunflowers form a distinct and well-supported clade containing eleven species, including the widely-distributed species *H. annuus* and *H. petiolaris*. These species likely originated in allopatry, but their current ranges show considerable overlap. Cytological studies and genetic maps constructed from interspecific crosses suggest that chromosomal rearrangements have accumulated since the evolutionary separation of *H. annuus* and *H. petiolaris*. These species are also separated by differences in morphology, life history and habitat preference, and show poor pollen viability in hybrid offspring [17-19].

Although *H. annuus* and *H. petiolaris* are estimated to have diverged from each other nearly 2 million years ago (Sambatti et al. 2012), they have been observed to hybridize in natural settings [20,21]. Average divergence between *H. annuus* and *H. petiolaris* is estimated to range from Fst = 0.19 (based on microsatellite variation) to Fst = 0.3 (based on sequence polymorphism in transcripts), similar to levels of intraspecific divergence among stickleback populations and between human populations from West Africa and East Asia [22-24]. This relatively low divergence is consistent with analyses of single-gene phylogenies that suggest substantial recent introgression between *H. annuus* and *H. petiolaris*[22]. In at least three cases, hybridization between *H. annuus* and *H. petiolaris* has led to the formation of distinct hybrid species (*H. anomalus*, *H. deserticola*, and *H. paradoxus*), which occupy extreme habitats (active sand dunes, desert, and salt marshes respectively). It has been hypothesized, with experimental support, that hybrids bearing genotypes associated with phenotypic traits and environmental tolerances outside of the range exhibited by either parental species were able to colonize unusual ecological niches and form new species [25,26].

Hybrids between *H. annuus* and *H. petiolaris* have also been created for research and agricultural purposes. Most prominently, *H. petiolaris* is the source of cytoplasmic male sterility PET1, widely used in commercial sunflower hybrid production [27]. *H. petiolaris* is a potential source of useful germplasm for improvement of *H. annuus* cultivar resistance to stresses, particularly osmotic stresses such as drought and saline soils.

Here we investigate patterns of transcript accumulation in hybrid sunflowers generated from controlled crosses of *Helianthus annuus* (cmsHA89) with *H. petiolaris* (Pet2152). We find that the majority of transcripts accumulate to intermediate levels in the F1 hybrid, and moreover, that mean transcript levels across parental accessions are highly predictive of transcript levels observed in F1 hybrids. Few transcripts showed accumulation outside of the range observed in parental accessions. Within F1 individuals, bias in accumulation of parental alleles was detected in 20% of transcripts where parental alleles could be reliably distinguished, but the magnitude of differences in accumulation were generally lower than differences observed between parental accessions. These results suggest that both *cis* and *trans* regulatory divergence contribute to interspecific differences in transcription, yet *H. annuus* and *H. petiolaris* genomes show relatively few instances of “misregulation” or extreme phenotypes at the transcript level.

## Methods

### Plant growth and generation of H. annuus × H. petiolaris hybrids

We used a cultivated accession of *H. annuus*, rather than a wild accession that might more closely represent the parents of homoploid hybrid sunflowers, for several practical reasons, described below: (1) Male sterility and distinct morphology of the *H. annuus* cultivar used, cmsHA89, provided better recovery and identification of hybrids than would be expected from crosses involving wild *H. annuus*. (2) cmsHA89 is not self-incompatible, but requires a pollen donor to produce viable seed. This reduced the chances of self fertilization due to mentor effects in mixed pollen loads (Desrochers et al. 1998). cmsHA89 sterility is conferred by the PET1 cytoplasm; while the ultimate origins of this cytoplasm remain unclear, it was introduced into *H. annuus* cultivated lines via introgression from *H. petiolaris*. However, the cmsHA89 cytotype is extremely rare in natural populations of Helianthus (Rieseberg et al. 1994). (3) The large heads of *H. annuus* cultivars provide greater potential seed yield per single cross than wild accessions. (4) The relatively homozygous genome of cmsHA89 also provided greater power to identify variants between parental genomes and assign parentage to alleles within hybrid offspring. This last factor is particularly important as *H. petiolaris* is intolerant to inbreeding and inbred lines of *H. petiolaris* are not available.

Plants of *H. annuus* cultivar cmsHA89 (USDA PI 650572) and wild *H. petiolaris* accession Pet2152 (USDA PI 586920) were grown in one gallon pots under standard greenhouse conditions at the University of British Columbia Botanical Garden Nursery. Before they began to open, cmsHA89 flowers were covered with drawstring organza bags to deter unauthorized pollination. When the anther filaments of at least the outer three rings of florets were exposed, cmsHA89 flowers were pollinated with pollen from a single Pet2152 plant and re-covered. Reciprocal crosses were not performed because cmsHA89 does not produce pollen. At the same time, self-incompatible Pet2152 plants were intercrossed. Seed heads were allowed to mature and dry before removal from the plant.

While crosses between *H. annuus* and *H. petiolaris* are generally of poor fertility, one cmsHA89 × Pet2152 cross produced approximately 200 mature seeds. F1 seeds (n = 60) were scarified (removal of the top 1/3 of the seed) to improve synchrony of germination and placed on moist filter paper disks in plastic petri dishes in the dark at approximately 25°C. cmsHA89 and Pet2152 (half-siblings of the F1 seedlings) were treated similarly. After 3 days, seedlings were transferred to soil in 32-cell nursery flats in a controlled environment chamber (16:8 light:dark, 50% RH, 28°C). After 3 additional weeks, plants were transplanted into 1-gallon pots and moved to a greenhouse bench.

The hybrid identity of putative F1 plants was confirmed via examination of external phenotypes and molecular markers. Visible phenotypic markers included pigmentation at the base of the stem, leaf shape, plant branching, and production of foliar glandular trichomes. Molecular marker phenotypes were observed by extraction of genomic DNA and amplification of two loci previously determined to differ in size (distinguishable by agarose gel electrophoresis) between cmsHA89 and Pet2152. PCR primers, amplification conditions, and representative gel images are provided in Additional file 1: Supplemental Methods 1.

### mRNA extraction and sequencing

At 45 days post-germination, leaf tissue was collected from 8 F1 plants and 2 plants from each parent accession. The youngest fully-expanded leaf was cut from each plant, placed into a 50 ml conical tube, and immediately frozen in liquid nitrogen. Total RNA was extracted from approximately 50 mg of ground tissue as described [28]. Preparation of non-normalized cDNA libraries and whole transcriptome shotgun sequencing (RNA-Seq) via Illumina HiSeq 2000 were performed at the Michael Smith Genome Sciences Centre in Vancouver, British Columbia, Canada (http://www.bcgsc.ca/services). Samples were multiplexed with 3 samples per lane.

### Sequence data processing and analysis

**Figure 1:**
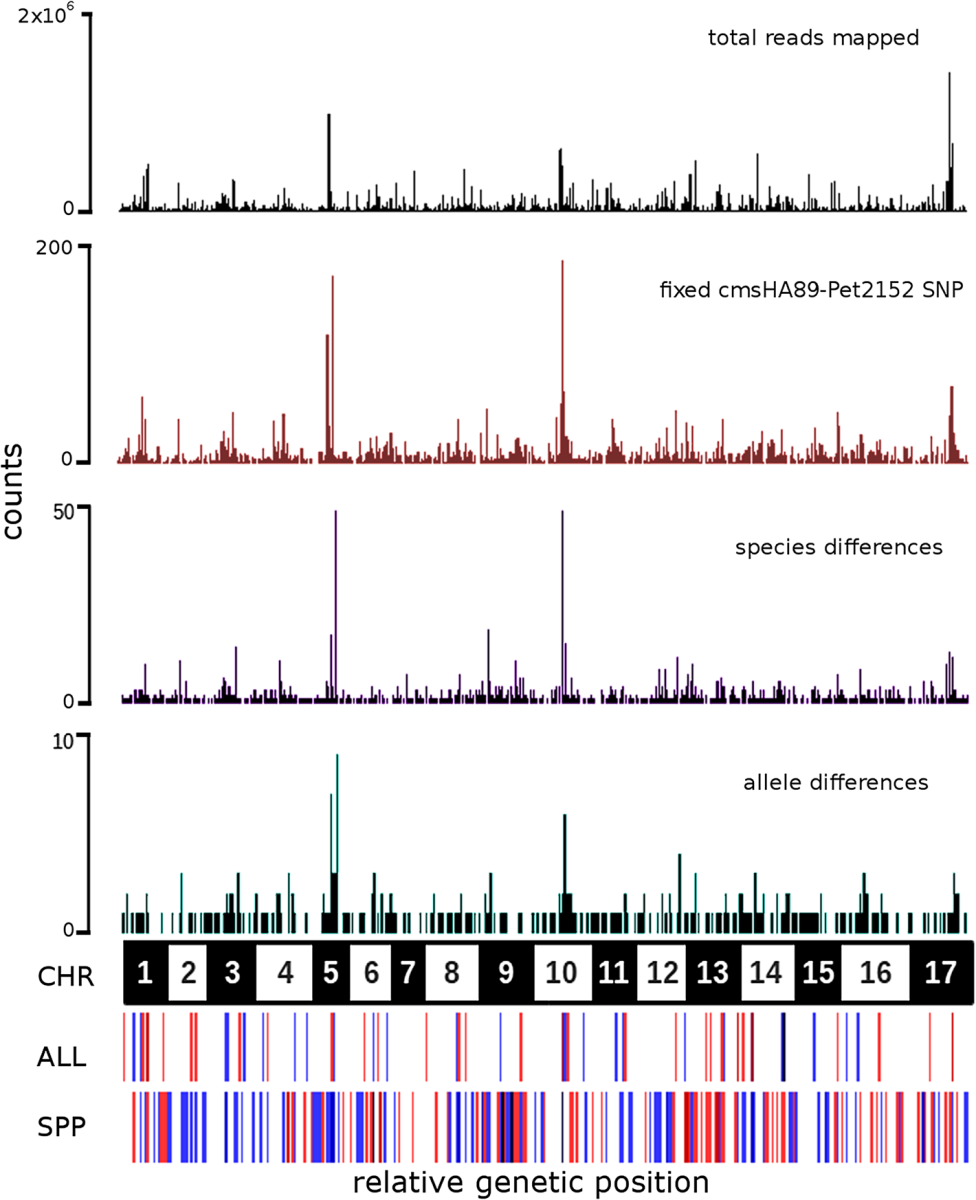
**Allelic bias in transcript accumulation within F1 hybrid plants.** The x-axis indicates genetic position along consecutively ordered chromosomes of the *H. annuus* genome (Additional file 2: Table S3); chromosome borders are delineated in the black and white bar labeled “CHR”. “total reads mapped” provides the sum of sequence reads (among 12 samples) assigned to each position. “fixed cmsHA89-Pet2152 SNP” identifies the location of variants used to assign allelic origin (see Methods). “species differences” shows location of contigs showing significant differences in transcript accumulation between *H. annuus* and *H. petiolaris* samples. “allele differences” shows the position of contigs identified as showing significant differences in accumulation of parental alleles in F1 hybrid samples. Bars labeled “ALL” and “SPP” show the summed direction of significant parental differences or allelic bias for that genetic map location; red indicates that the *H. annuus* samples or alleles show higher transcript accumulation, blue indicates that *H. petiolaris* samples or alleles show higher transcript accumulation.

Paired-end, 100bp RNA-Seq reads (chastity > 0.6) were aligned to a *H. annuus*-derived transcriptome reference [29]. This reference, assembled from 93428 EST sequences [30,31], consists of 16312 unique contigs with a total length of 17.062 million bases. Fasta-formatted sequence for the transcript reference is available at datadryad.org [29]. The median insert size between paired-end reads ranged from 131 to 151 bases per sample. Approximately 52% (8559) of reference contigs were assigned to genetic map positions within the *H. annuus* genome via identity to sequenced markers appearing on a map of *H. annuus* derived from recombinant inbred lines from the population RHA280 × RHA801 (Renaut et al. *in review,*[32]) (Figure 1). Genetic map positions assigned to the transcript reference are available as Additional file 2: Table S3. Alignments were performed using the Burrows-Wheeler Aligner (BWA) tools ‘aln’ and ‘sampe’ using a maximum insert size of 1000 and a quality filter of 30 to trim reads [33]. Aligned BAM files were sorted and PCR duplicates removed using SAMtools utilities ‘sort’ and ‘rmdup’ [34]. Reads per contig were counted for each sample using coverageBed [35].

Read counts were analyzed in R using the DESeq package to compare counts of reads aligned to a given reference contig [36]. The DESeq package uses a modified Fisher’s exact test with data fit to a negative binomial distribution to test for pair-wise differences in count data between sample classes, allowing within-transcript comparisons across a broad dynamic range. Three pairwise comparisons were performed to identify contigs that showed differences in accumulated mapped transcript reads between *H. annuus* cmsHA89 and *H. petiolaris* Pet2152, cmsHA89 and F1 samples, and Pet2152 and F1 samples.

Per-contig read counts were averaged across parental accession samples (cmsHA89, Pet2152) to generate mean parent values, and across samples from F1 hybrid plants. Linear modeling of mean transcript counts from F1 plants as a function of mean parent transcript levels was performed in R. Examination of residuals and leverage estimates for this model led us to remove 3 contigs with values of Cook’s D exceeding 1. Refitting the model without these points did not significantly alter the parameter estimates for the model. Predictions of hybrid transcript values, with 99% confidence intervals, were generated using this model. Reference contigs showing mean hybrid transcript accumulation outside the confines of the confidence interval for predictions were classed as “non-additive”.

### Variance among hybrids

Variability in transcript levels among individual F1 hybrids was assessed by calculating the coefficient of variation (CV) for each reference contig. To reduce bias in the estimates of CV due to non-normal distribution of transcript level estimates, read counts were first subjected to a natural log transformation and CV was calculated using the formula CV = sqrt((e^σ(ln)^)^2^ - 1), where σ(ln) is the sample standard deviation calculated from the log-transformed hybrid transcript values. Contigs with a CV greater than 2 were considered to have high variance among hybrid plants.

### Allelic bias in transcript accumulation

Data from the four sequenced parental accession samples (HA89.5, HA89.9, PET.2, PET.3) were analyzed simultaneously using SAMtools ‘mpileup’ to identify single nucleotide polymorphisms (SNPs) with respect to the reference sequence. We used a custom perl script to extract loci meeting the following criteria: 1) the variant allele frequency ≠ 1 (this criterion excludes sites where samples differ from the reference, but not between accessions), 2) phred quality score ≥ 80, 3) a single allele is detected within each accession, and 4) the number of sequence reads covering the position is ≥ 5 for each sample (Additional file 3: Supplemental Methods 2). This final criterion eliminates potential false discovery of allelic bias due to failure of one parental allele to align to the reference transcript set, yet also eliminates sequences that are not transcribed (at a detectable level under our conditions of growth and sampling) in one parent genome that may show true allelic bias in the F1 offspring.

For each qualifying variant position within a contig, read depth per SNP was determined for both *H. annuus* and *H. petiolaris*-derived variants within individual F1 transcript sequence datasets. From SAMtools ‘mpileup’ output for individual hybrid plants, we extracted ‘dp4’ (read depth for: reference allele on the forward strand, reference allele on the reverse strand, alternate allele on the forward strand, alternate allele on the reverse strand) at each target site, and combined forward and reverse read counts to determine per-allele read depth. At this stage we also removed variants that were only detected on one direction of sequence read (either forward or reverse), as these are likely to represent sequencing artifacts. Read counts for each variant were compared across F1 samples using DESeq [36]. For later gene-level analyses, significant SNP within the same contig were considered as a single significantly-differing transcript, with allelic bias estimated as an average of differences in read counts per SNP and positions showing inconsistent results (i.e. one position shows significant bias toward the *H. annuus* variant, while the other shows bias toward the *H. petiolaris* variant) flagged. To assess the general level of transcript level variation due to *cis* regulatory divergence between parental genomes versus *trans*-acting regulators, we examined the overlap between reference contigs with one or more fixed SNP showing significant differences in transcript accumulation between parental accessions and those showing significant allelic bias in F1 hybrids. We also fitted a linear model to predict the magnitude of allelic bias based on the observed difference in transcript level between parental accessions.

### Classification and annotation of transcripts

Contigs with transcript accumulation patterns suggesting non-additive interactions between parental genomes within hybrid individuals, as revealed by the analyses described above, were labeled as ‘non-additive’, ‘transgressive’, ‘high variance’, or ‘allelic bias’. We identified non-additive transcripts as those showing significant deviation of mean transcript levels in hybrid plants from combined mean transcript accumulation of parental accessions (e.g. those hybrid transcript values falling outside the 99% confidence interval of the linear model associating hybrid transcript with mean parent transcript levels). Transcripts labeled ‘transgressive’ showed mean accumulation within the F1 hybrids that was significantly greater or less than the mean values observed for both *H. annuus* and *H. petiolaris*. While transgressive levels of transcript accumulation should also be described as non-additive, these two categories do not fully overlap due to the differences in analyses used to define them. Situations where the difference between *H. annuus* and *H. petiolaris* is large or there is variation in transcript abundance within parental accessions may broaden the confidence interval encompassing ‘additive’ values for F1s, despite F1 means significantly differing from both parents. ‘High variance’ contigs showed estimates of the coefficient of variation across F1s that were greater than 2. The set of reference contigs labeled ‘allelic bias’ contained at least one SNP that distinguished the two parental alleles (see criteria above) with variants represented in mapped cDNA sequence reads at a ratio significantly different from equality.

Potential functions of reference transcript contigs identified as non-additive in F1s according to any of the above criteria were explored via analysis of similarity to published protein (NCBI non-redundant protein RefSeq [37]) and nucleotide databases using blastx and blastn from NCBI-BLAST + (http://www.ncbi.nlm.nih.gov/books/NBK1763/), filtering results with e-values greater than 1e-10. Analyses of gene ontology (GO) for contigs of interest were performed using GOrilla, with refinement via ReviGO [38,39]. For each gene list, contigs showing significant similarity to *Arabidopsis thaliana* TAIR10 sequences with GO annotations were compared to three separate similarly-sized lists of contigs randomly drawn from the *H. annuus* reference transcript set using a hypergeometric distribution (modified Fisher’s Exact Test). GO processes found to be significantly (FDR-adjusted p-value < 0.05) over-represented in all three analyses are discussed.

## Results

F1 seeds derived from fertilization of *H. annuus* cmsHA89 with *H. petiolaris* Pet2152 pollen germinated with 88% success. F1 plants exhibited intermediate phenotypes with respect to parental accessions for all quantitative traits measured, except days to flowering, where F1 plants flowered, on average, earlier than plants of either parental accession (Table 1). F1 plants showed 1:1 segregation in production of pollen, suggesting that the Pet2152 pollen parent was heterozygous for a nuclear fertility-restoring locus complimentary to the cytoplasmic male sterility present in cmsHA89.

**Table 1:** **External phenotypes of *H. annuus *cmsHA89, *H. petiolaris *Pet2152, and F1 hybrid offspring**

	**Pollen**	**Days to flowering**^**a**^	**Number of flowers**^**b**^	**Flower diameter (mm)**^**c**^	**Trichome density**^**d**^	**Branching**^**e**^
*H. annuus* cmsHA89	N	67.3 (2.0)	1.2 (0.7)	43.3 (12.3)	437.4 (180.5)	0 (0)
*H. petiolaris* Pet2152	Y	62 (6.2)	48.1 (7.3)	15.8 (2.5)	0 (0)	8.7 (1.2)
F1 (mean)	Y/N	54.4 (2.0)	15.5 (7.2)	29.3 (4.6)	48.3 (40.9)	5.9 (1.7)
F1.01	N	52	11	32.3	31.5	7
F1.03	N	52	22	30.2	59.3	8
F1.04	N	55	5	29.2	25.9	5
F1.07	Y	55	14	28.5	53.7	7
F1.14	N	55	11	26.9	36.1	6
F1.17	Y	53	16	25.8	25.9	7
F1.TA	Y	54	17	21.6	51.9	6
F1.TB	N	55	17	24.8	11.1	4

Data provided are mean values (standard deviation). Phenotypes for individual F1 plants sampled for transcript sequencing are provided below the mean values for the total (n = 52) F1 plants grown. ^a^number of days post-germination when the first flower opened; ^b^number of flowers produced by the plant; ^c^disk diameter for the first flower (does not include ray flowers); ^d^foliar glandular trichomes per cm^2^ leaf abaxial surface; ^e^total number of lateral branches > 2 mm in thickness.

RNA extraction and Illumina shotgun sequencing of cDNA were performed for eight F1 plants as well as two plants from each parental accession, generating an approximate average of 27 million 100 bp paired-end reads per sample. Linear modeling of sequence output showed no significant difference among accessions in the number of reads generated per sample (F = 0.0728, p = 0.93, R^2^ for the model = 0.0159). However, a significantly smaller percentage of Pet2152 reads were successfully mapped to the *H. annuus*-derived reference transcript dataset when compared to HA89: 51.94 (± 0.90) vs. 58.26 (± 0.37) percent mapped, F = 4.826, p(model) = 0.037, p(HA89-Pet2152 ≠ 0) = 0.013, R^2^ for the model = 0.5175. Sequence reads obtained from F1 hybrid plants mapped to the reference with intermediate success: 55.05 ± 2.27 percent mapped, p(HA89-F1 ≠ 0) = 0.077. Of 16312 contigs contained in the reference transcript set, approximately 2.5% had no reads mapped from the combined 12 samples and 7.5% (1220 contigs) had a per-sample average depth of less than ten sequence reads.

Examining the relationships among samples for transcript accumulation levels over the entire transcript reference via both Spearman correlation and principle components analyses showed that two samples, HA89.9 and F1.TA, grouped together rather than with other HA89 or F1-derived samples. The average coefficient of the pair-wise correlation between F1.TA and other F1 samples was 0.818, while the range of correlation coefficients (R^2^) for comparisons among F1 (excluding F1.TA) was 0.977-0.998. Similarly, while cmsHA89 samples were significantly and positively correlated (R^2^ = 0.755), transcript levels showed higher similarity between HA89.9 and F1.TA (R^2^ = 0.977). As patterns of sequence polymorphism, in addition to earlier genotyping, confirmed that these samples were identified correctly, we hypothesize that uncontrolled environmental factors influenced transcript accumulation patterns in these two plants, despite our attempts to maintain similar conditions. In particular, when compared to all other samples these plants show relatively reduced accumulation of transcripts involved in photosynthetic processes, and relative increases in accumulation of transcripts associated with defense and stress responses. To avoid excessive influence of these samples on interpretation of our data, we conducted analyses of both the complete data (n = 12) and a dataset from which HA89.9 and F1.TA had been removed (n = 10). Results presented below show the overlap between these two analyses; differences between analyses of complete and reduced datasets are shown in Additional file 4: Figure S1.

### Interspecific differences in transcript accumulation

**Figure 2:**
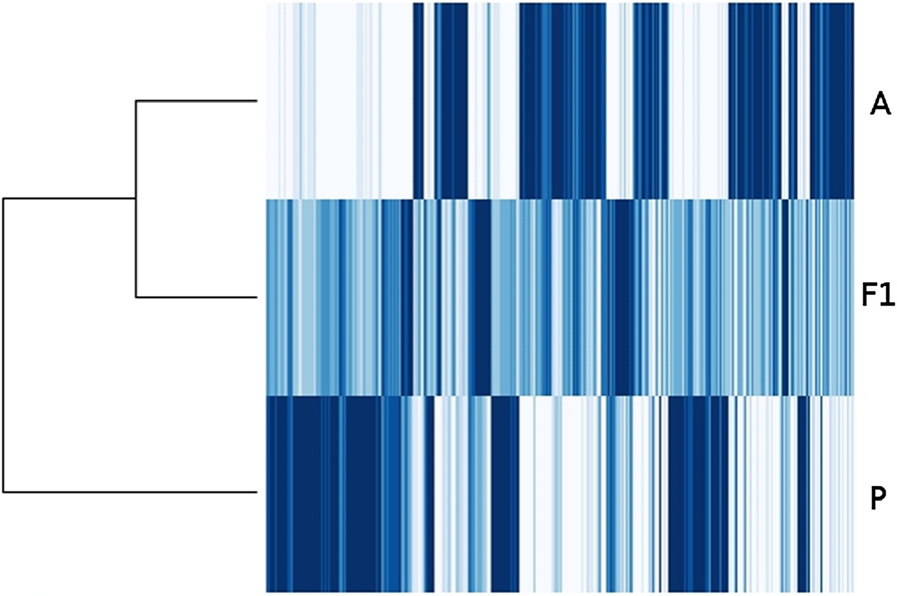
**Heatmap of z-score normalized means of mapped read counts for 1488 genes significantly differing in transcript accumulation (q-value < 0.01) for at least one pairwise comparison among *H. annuus *cmsHA89 (A), *H. petiolaris *Pet2152 (P), and cmsHA89 x Pet2152 F1 interspecific hybrids (F1).** Only transcripts with significant differences conserved between full and reduced analyses are shown. Accession groups are shown as columns. Individual transcripts are arrayed in rows; a list of reference identifiers, mean read counts, and annotation by similarity to published sequences is provided as Additional file 5: Table S1. Shading indicates lower (lighter) or higher (darker) relative transcript values.

Differential transcript accumulation was assessed via pair-wise comparison of transcriptome shotgun sequence from *H. annuus* cmsHA89, *H. petiolaris* Pet2152 and cmsHA89 × Pet2152 F1 hybrids. Per-contig transcript accumulation, measured as mapped sequence reads per contig, differed between accessions for 1456 (cmsHA89 vs. Pet2152), 125 (cmsHA89 vs. F1), and 1555 (Pet2152 vs. F1) transcripts, using a q-value (adjusted for multiple comparisons) of 0.01 (Figure 2; Additional file 5: Table S1). High variance between the cmsHA89 samples (discussed above) likely contributes to the lower number of significantly-differing transcripts detected in comparisons involving this accession. A greater number of contigs (64.7%) showed elevated transcript accumulation in cmsHA89 (943 contigs) versus Pet2152 (513 contigs). These included 169 contigs with no reads mapped from one accession, with 123 of these containing no mapped transcript reads from Pet2152. A similar bias was observed in comparisons of cmsHA89 to F1 hybrids, as 80 of 125 contigs significantly differing in transcript accumulation (64%) showed elevated counts in HA89 samples. However, comparison of F1s with Pet2152 showed greater similarity in the numbers of transcripts elevated for each accession, with approximately 54% (835 contigs) showing higher transcript accumulation in *H. petiolaris* samples relative to F1 samples.

### Non-additive transcript accumulation in F1 hybrids

**Figure 3:**
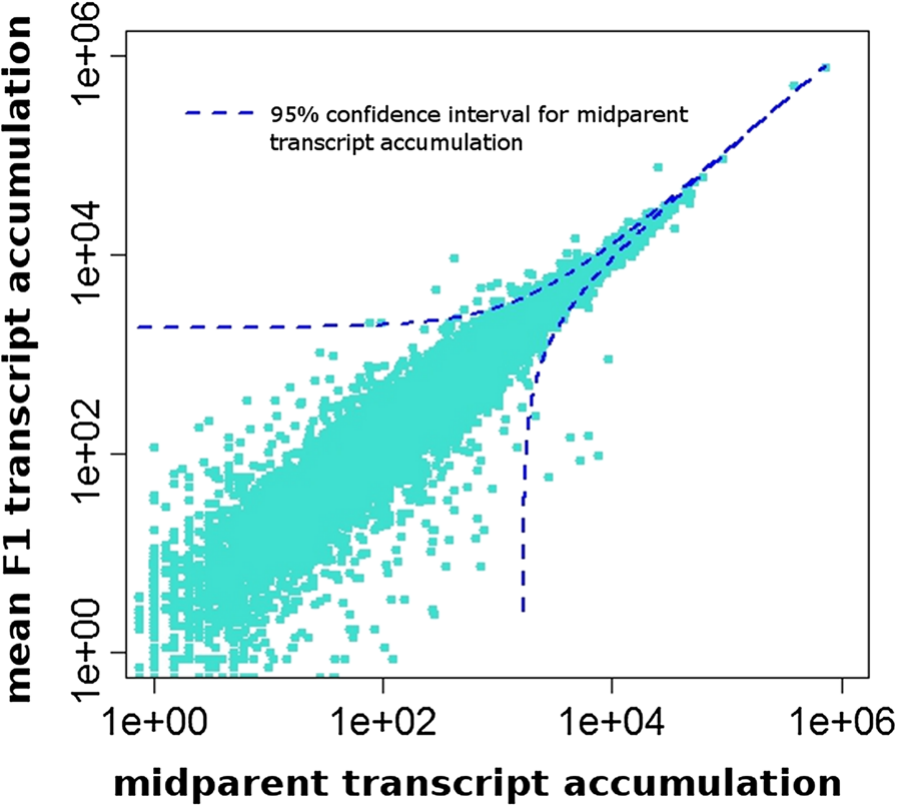
**Combined mean transcript accumulation (count of mapped reads) for parental accessions *H. annuus *cmsHA89 and *H. petiolaris *Pet2152 plotted on the horizontal axis against mean transcript accumulation in F1 hybrid plants (vertical axis).** Each point represents one of 16,312 contigs in the reference transcript set. Values on both axes are plotted on a log_10_ scale. Dotted lines indicate the 99% confidence interval for F1 hybrid transcript accumulation predicted by the linear model: F1 transcript accumulation = SLOPE*mean parental transcript accumulation + INTERCEPT (p < 0.0001, R^2^ = 0.98).

F1 plants showed intermediate levels of transcript accumulation for more than 99% of these comparisons (Figure 3). A linear model of mean transcript accumulation across F1 plants as a function of the mean of parental samples explained a high proportion of F1 transcript variance (p < 0.0001, R^2^ = 0.98 (reduced data), p < 0.0001, R^2^ = 0.96 (full data)). Slope and intercept were estimated as 1.09 (±0.0011) and 2.278 (±7.384) (reduced dataset), respectively. Modeling transcript accumulation for individual F1 plants against midparent values generated model R^2^ values ranging from 0.908 to 0.947, with the exception of the outlier sample F1.TA, where midparent values explained only one third of transcript level variance. Only 159 contigs had mean hybrid read counts outside the 99% confidence intervals generated for predicted hybrid transcript values in both analyses (Additional file 5: Table S1). The mean transcript accumulation estimates for these contigs in F1 plants ranged from 1.2 to 2372% of predicted values, roughly evenly divided between those above (44%) or below (56%) the parental mean.

Analysis of gene ontologies (GO) assigned to these transcripts indicated significant overrepresentation of 160 GO processes, reduced to 64 by collapsing highly redundant categories. Among these GO terms, two groups were prominent, involving photosynthesis and energy metabolism (including photosystem assembly, chlorophyll biosynthesis, plastid localization, pentose-phosphate shunt, electron transport) and defense response (including salicylic acid biosynthesis, regulation of hypersensitive response, MAPK cascades, jasmonate signaling).

### Transgressive transcript accumulation in F1 hybrids

While the majority of contigs examined showed intermediate levels of transcript accumulation in F1 plants relative to parent accessions, 10 contigs consistently showed transcript accumulation significantly greater or less than values observed in cmsHA89 or Pet2152 samples (Table 2). Of these, 8 transcripts showed higher accumulation in hybrids than parental accessions, a bias that is maintained when the criteria for identifying contigs as significantly transgressive are relaxed to include contigs significant in only one of the two analyses (full or reduced dataset): 52/65 identified contigs showed higher transcript accumulation in F1s under these relaxed criteria (Additional file 6: Table S2). In addition, 50 reference contigs identified as showing non-additive F1 transcript accumulation had F1 mean values either higher (41) or lower (9) than both parent mean values (Additional file 5: Table S1).

**Table 2:** **Contigs showing significantly transgressive transcript phenotypes across F1 *H. annuus *x *H. petiolaris *hybrids in both full and reduced analyses**

**ContigID**	**A_full**	**H_full**	**P_full**	**A_red**	**H_red**	**P_red**	**Transgressive**	**Summary annotation**
BigSet000391	29	4	36	13	4	36	LOW	beta-glucosidase
BigSet003246	44	4	28	68	4	28	LOW	carboxy-lyase
BigSet010982	1	47	1	0	52	1	HIGH	cuticular wax
BigSet009445	4	50	0	8	6	0	HIGH	oxylipin biosynthesis
BigSet011026	1	15	0	1	12	0	HIGH	not_informative
BigSet015125	11	89	10	20	48	10	HIGH	protease inhibitor
BigSet011931	0	69	4	0	50	4	HIGH	protease inhibitor
BigSet009443	4	27	2	7	31	2	HIGH	protein degradation
BigSet007352	5	122	7	3	139	7	HIGH	peptide transporter
BigSet015238	0	10	0	0	11	0	HIGH	unknown

(Reduced analyses omit outlier samples HA89.9, F1.TA.) Mean transcript accumulation (RPKM) per accession (A = *H. annuus* cmsHA89, H = F1 hybrid, P = *H. petiolaris* Pet2152) are provided for both full (“full”) and reduced (“red”) analyses. ‘summary annotation’ summarizes the hypothesized gene function based on BLAST-identified similarity. Details of BLAST analyses are provided in Additional file 6: Table S2.

### Variance among F1 hybrids

**Figure 4:**
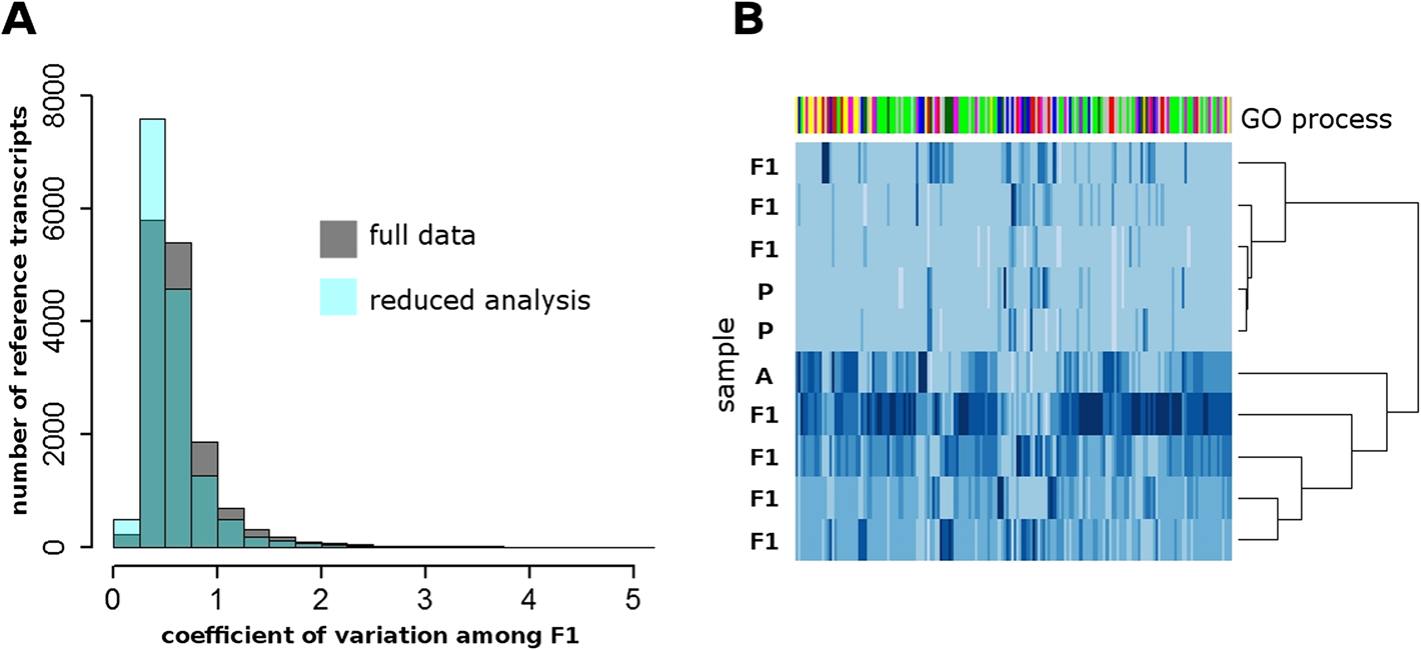
**Variation in transcript accumulation among F1 hybrid individuals. A**) Distribution of the coefficient of variation (CV) for 16,312 transcripts analyzed from F1 plants; grey = CV calculated from full data, light blue = CV calculated from reduced data (minus F1.TA), darker blue indicates overlap. **B**) Heatmap showing z-score normalized transcript accumulation for 166 reference contigs with CV > 2 within F1 samples. Samples from individual plants are shown in horizontal rows; F1 = hybrid F1, A = *H.annuus* cmsHA89, P = *H. petiolaris* Pet2152. Hierarchical clustering estimated from Spearman correlation coefficients for pairwise contig (x-axis) and sample (x-axis) distance matrices. The colored bar along the top edge indicates assignment of transcripts to GO Biological Process groups, with prominent categories: green (cell cycle/mitosis), yellow (histones/chromatin modification), blue (metabolism), red (stress/defense), and pink (transcription factors/signaling).

The F1 plants examined in this study are the product of hybridization between an inbred *H. annuus* domesticated line and a wild-collected *H. petiolaris* accession that is highly heterozygous. Both external and transcript-level phenotypes evaluated in F1 plants were largely intermediate with respect to the parental accessions, yet did show variation among F1s (Table 1). Transcript sequences were obtained from eight individual F1 plants, allowing us to evaluate inter-plant variation in transcript accumulation that may be attributable to interaction with segregating regulatory loci in a parental genome. We calculated the coefficient of variation for each transcript across all F1 plants (Figure 4A). 166 contigs (approximately 1% of the reference transcriptome) consistently had a CV greater than 2 for F1 samples (Additional file 5: Table S1). These mainly included genes controlling cell division and DNA synthesis. When these contigs are subjected to hierarchical clustering, F1 samples form one relatively uniform group resembling *H. petiolaris* samples, and one more variable group including *H. annuus* (Figure 4B, Additional file 7: Figure S2). This is consistent with segregation of parental alleles associated with regulation of these transcripts.

### Allelic bias in transcript accumulated in F1 hybrids

**Figure 5:**
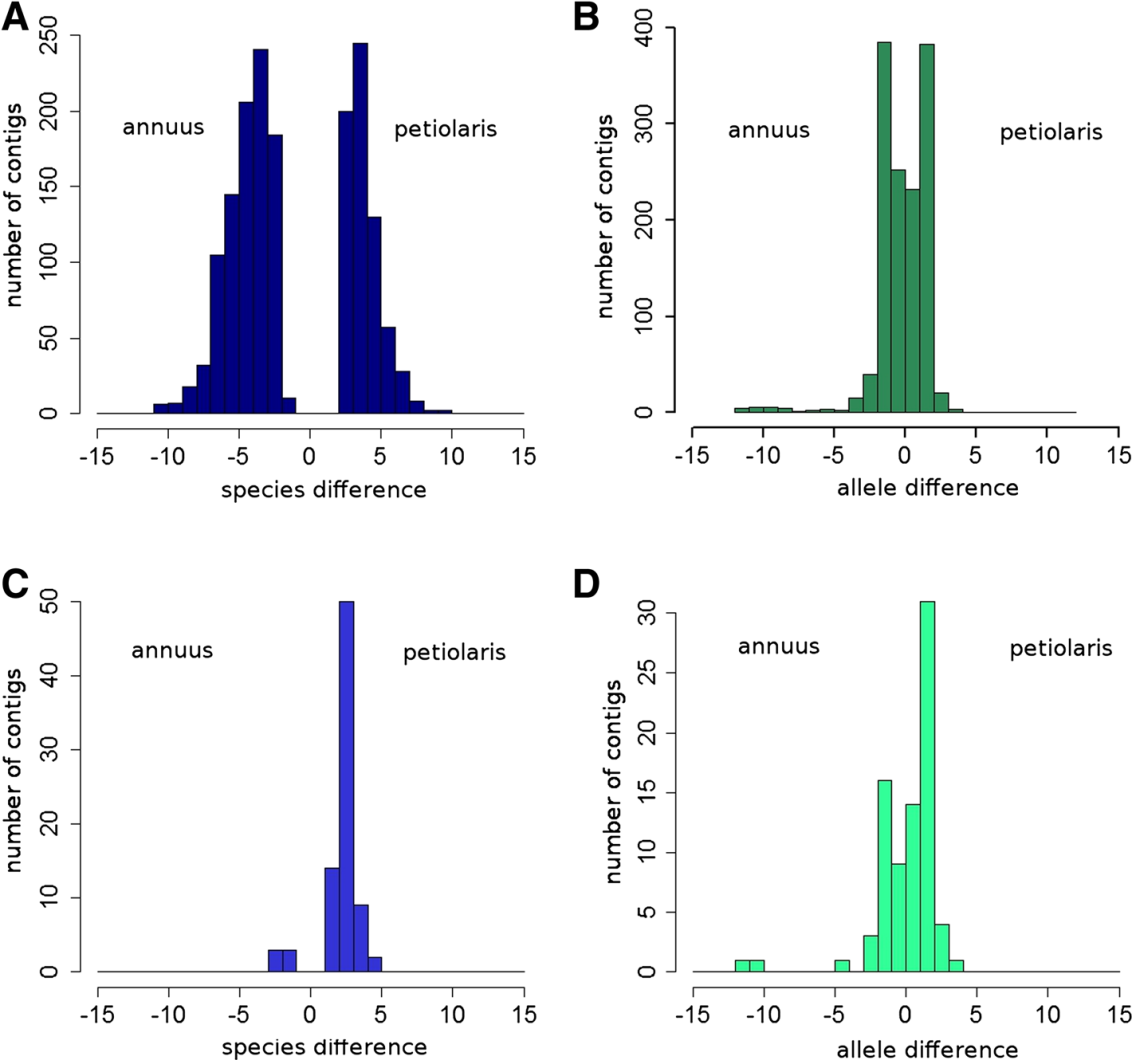
**Magnitude of significant transcript accumulation differences observed between parental accessions *H. annuus *cmsHA89 and *H. petiolaris *Pet2152 (A, C) and between parental alleles within F1 hybrids (B, D).** Horizontal axes show the log_2_ fold-change in transcript accumulation associated with a shift from *H. annuus* to *H. petiolaris*, thus positive values indicate relatively higher levels of transcript accumulation in *H. petiolaris* (**A**, **C**) or of the *H. petiolaris* allele within the F1 (**B**, **D**). Vertical axes show the number of contigs showing statistically significant differences between accessions or alleles (adjusted p-value < 0.01). Panels **A** and **B** show the distribution of all significant results, while panels **C** and **D** show only contigs from the reference dataset that show significant differences in transcript levels both between accessions and between alleles.

13,734 fixed single nucleotide variants (SNP) were identified between *H. annuus* and *H. petiolaris* transcript reads, contained within 3,393 contigs. These SNPs were distributed across the genome, with a high correlation between the density of SNPs detected and the overall abundance of sequence reads mapped to a given genetic position (Spearman correlation: R^2^ = 0.93) (Figure 1). It was therefore possible to distinguish between parent genome contributions to the F1 hybrid transcript pool for approximately 20% percent of the reference transcript set. The average Spearman correlation coefficient of transcript levels between alleles within individual F1 samples was 0.82 (±0.015). We identified 1,363 polymorphic sites within 681 contigs where allelic variants derived from the two parental genomes were detected in significantly different quantities, indicating allelic bias in transcript accumulation. For the majority of transcripts, the magnitude of the allelic bias is relatively small, with the dominant allele present at approximately twice the level of the alternate parental allele (Figure 5).

Of the 681 contigs containing at least one SNP showing significant bias in F1s, only 81 were also identified as showing significant differences in transcript accumulation between parental accessions cmsHA89 and PET2152 (Figure 5). A conservative estimate based on overlap of both full and reduced analyses indicates that 1456 (or 9% of) reference transcripts examined differ in transcript accumulation between parental accessions. Differences between parental accessions were consistent with differences between alleles in 65% of the contigs showing significant differences in both sets of analyses. All inconsistent contigs showed significantly higher levels of accumulation in *H. petiolaris* (compared to *H. annuus*) samples but significantly lower accumulation of transcripts bearing *H. petiolaris* alleles in samples from hybrids. Across all contigs showing significant evidence of allelic bias, almost 77% (523/681) showed higher transcript levels in *H. petiolaris* samples than *H. annuus* samples, suggesting that the larger number of trancripts observed showing bias toward the *H. annuus* allele in hybrid samples is not simply explained by preferential alignment of transcript sequence reads to the *H. annuus*-based reference transcriptome.

## Discussion

Gene expression changes associated with hybridization may be attributed to a variety of factors. Novel gene combinations, chromosomal rearrangements, increases in transposon activity, and changes in DNA methylation status occur in interspecific hybrids and are likely to affect gene expression [30,31,40-43]. The transcriptional phenotypes of first generation hybrids should predominantly reflect the basic interaction of parental genomes and their endogenous regulatory factors. Deviation of F1 transcript accumulation from midparent values (expected if parental genome contributions to hybrid transcript accumulation were purely additive) will reflect epistatic and dominance interactions between parental genomes.

The transcript patterns observed in annual sunflower hybrids in this study differ from other systems used to study homoploid hybridization in experimental settings, such as *Drosophila* interspecies hybrids and re-synthesized *Senecio squalidus*, where relatively high proportions of transcripts examined showed “misexpression” or allelic bias [14,15,44]. Various lines of evidence suggest that *H. annuus* and *H. petiolaris* have experienced substantial levels of recent genetic exchange, in several instances resulting in ecologically mediated formation of hybrid species [22,25,45-48]. While reduced divergence through introgression might be expected to increase genomic compatibility, selection for hybrid viability should also select against extreme levels of genomic misregulation. In this study, we have selected not merely for strict viability, but for growth beyond the seedling stage. It remains possible that regulatory incompatibilities have greater impact on early stages of growth and development, or specifically in reproductive tissues, and thus are not detected in this study, which, as is generally true for analyses of transcript accumulation, can only provide a snapshot of the continuous flow of transcript production and degradation. In this experiment, we also observed strong, uncontrolled environmental effects on transcript profiles that led to a loss of experimental power, most prominently affecting our ability to confidently identify transcriptional differences between *H. annuus* cmsHA89 and *H. petiolaris* PET2152 or F1 hybrids. Comparisons between *H. petiolaris* and F1, or within F1, are relatively unaffected. While this means that we may underestimate transcriptional divergence of F1 from the maternal parent, a broader implication is that uncontrolled environmental factors can have dramatic effects on transcription. The distribution of random effects within the generally resource-limited designs of many transcriptional profiling experiments may have profound effects on the conclusions drawn from these experiments, which would be exacerbated by genotype by environment interaction.

It is believed that formation of *Helianthus* hybrid species has been mediated by environmental selection on transgressive phenotypes generated through segregation of parental genomes [25,26,28]. At the same time, interacting parental genomes present in early generation hybrids must generate phenotypes with sufficient fitness to survive beyond the initial hybrid generation for novel segregants to appear. Naturally-occurring hybrid individuals, as well as laboratory-derived first-generation hybrids, appear to exhibit intermediate phenotypes for many morphological and phenological traits (Table 1) [19-21,49]. This study suggests that *H. annuus* × *H. petiolaris* F1 hybrids also exhibit quantitatively intermediate phenotypes at the level of transcript accumulation, reflecting widespread compatibility between diverged parental transcript regulatory networks. The small sample sizes for parental accessions in this study may have hindered detection of transgressive transcription in F1 hybrids, through increased uncertainty regarding actual parental transcript levels. Our approach still provides an improvement in estimating parental transcription over strategies employing pooled samples, and focusing sampling effort on individual F1s has provided more reliable estimates of both the mean and variance of transcript levels in hybrids.

Although *Senecio aethnensis* and *S. chrysanthemifolius* (the parents of the homoploid hybrid *S. squalidus*) form a well-established hybrid zone with evidence of substantial gene flow between species, a much larger percentage of the analyzed transcript of first generation hybrids showed evidence of non-additive (4.9%) or transgressive (3.2%) accumulation [14,50,51]. The relative scarcity of non-additive (0.97–1.28%) or transgressive (0.06–0.7%) transcriptional phenotypes in this study might be attributed to differences in methodology. Quantification of transcript levels via sequencing techniques, rather than hybridization-based microarray platforms, allow both examination of a broader array of transcripts and greater sensitivity to detect low-level transcripts (without necessarily increasing statistical power to detect differences in these transcripts). Analysis of individual F1 plants allowed assessment of transcript variance among hybrids; distributions of hybrid transcript levels may suggest different relationships to parental transcript levels than values generated from pooled hybrid samples. Differences in the historic patterns of hybridization and selection, or in the phylogenetic distance between hybridizing species (much greater for *H. annuus* and *H. petiolaris* than between the two sister *Senecio* species in question), might also account for the different outcomes observed in these two hybrid systems. In particular, the female parental lineage of the hybrids examined in this study is a product of modern breeding, which has included hybridization with wild sunflowers [52].

Less than 1% of analyzed hybrid transcripts levels fell outside the predictive 99% confidence interval based on averaged transcript levels from parental accessions (Figure 3). Thus, non-additive transcript levels in these F1 plants are detected at a frequency indistinguishable from that expected by chance. These transcripts do, however, show significant over-representation of transcripts predicted to function in the broad categories photosynthesis/energy metabolism and response to biotic stimulus. In particular, transcripts participating in photosynthetic and energy processes are likely to be influenced by interaction with cytoplasmic components, even if the genes themselves are transmitted through nuclear inheritance. These transcripts also accumulate to high levels, potentially increasing the relative statistical power to identify variance from expected transcript values. The GO terms associated with the group of non-additively accumulated transcripts putatively involved in responses to biotic stimuli include defense response to bacterium, salicylic acid biosynthesis and metabolism, systemic acquired resistance, and MAPK cascade signaling. Misregulation or allelic incompatibility of genes involved in plant immune responses, particularly related to specific recognition of biotrophic pathogens, has been implicated in hybrid necroses (an extreme example of hybrid genome incompatibility) in *Arabidopsis thaliana*, lettuce, and wheat [53-55]. The hybrid plants in this study showed no obvious sign of hybrid necroses under relatively benign growth conditions, and rigorous examination of the phenotypic consequences of altered transcript levels for these immunity-associated genes will be necessary to determine whether immune incompatibilities are likely to have significant evolutionary consequences for *Helianthus* hybrids.

Interspecific hybridization presents the opportunity to distinguish the effects of nucleotide sequence variation associated with the transcript site (*cis* variation) and polymorphism in *trans*-acting regulatory factors. Variation in transcript accumulation between parental accessions that is caused by polymorphism in *trans*-acting factors should be diminished in hybrid individuals where transcription factors from both genomes are present. The allelic bias detected in F1 hybrids suggests that many differences observed between parental accessions are attributable to *cis *variation, although the magnitude of allelic bias is generally smaller than the difference in transcript levels observed between parental accessions. The observed expression patterns might therefore be a product of regulatory interaction within or between loci.

Analyses of gene ontology indicated that the group of transcripts showing significant allelic bias is enriched for processes including chloroplast organization, energy metabolism, translation, rRNA processing, and biosynthesis of isopentenyl diphosphate via the non-mevalonate (plastid-based) pathway. As these processes all involve cytoplasmically-inherited cellular components, it is plausible that nuclear-cytoplasmic interactions drive the allelic biases in transcript accumulation observed in hybrids. Despite *H. annuus* serving as the maternal parent of the hybrids, all over-represented gene groups examined contained a mixture of transcripts showing overrepresentation of *H. annuus* or *H. petiolaris* alleles.

The extent of *cis* regulatory differences between *H. annuus* and *H. petiolaris* transcripts is likely underestimated in the approach presented here. The criteria for selection of variants used to assign parentage to transcripts within F1 individuals excludes both loci lacking mapped transcript reads from either parental accession and loci that are polymorphic within either parental accession. While, on average, approximately 130,000 high-confidence heterozygous sites were identified per F1 individual, parentage could only be reliably assigned for a fraction of these. In addition, transcripts affected by polymorphism in *cis* regulatory sequences, but lacking consistent sequence polymorphism between parental accessions within the actual transcripts, will not be detected as showing allelic bias, although transcripts from such loci may be preferentially derived from one parental genome [16,56,57]. A relative lack of fixed polymorphism in transcripts showing expression differences between parental accessions contributes to the minimal overlap observed between transcripts showing allelic bias and those showing differences in accumulation between parental accessions. This suggests that *cis* and *trans* regulation are both influential in first generation hybrids, but confidently apportioning their relative effects will require additional data from non-coding regulatory regions.

## Conclusion

Studies typically focus on the extreme consequences of hybridization, both the good (heterosis) and the bad (genomic incompatibilities and hybrid necroses) [53-55,58-61]. This study, in contrast, detects few extreme transcript phenotypes in hybrid offspring of two annual sunflower species that show evidence of extensive gene flow since their divergence. Comparison of additional hybrid transcriptomes from crosses of wild sympatric and allopatric *H. annuus* and *H. petiolaris*, particularly incorporating a range of tissues and developmental stages, may clarify the role that introgression plays in transcriptional compatibility.

### Availability of supporting data

RNAseq data used in this study are deposited in the NCBI Sequence Read Archive under accession numbers SRS1993196 through SRS1993207.

## Abbreviations

CV: Coefficient of variation; EST: Expressed sequence tag; FDR: False discovery rate; GO: Gene ontology; MAPK: Mitogen-activated protein kinase; PCR: Polymerase chain reaction; RPKM: Reads per kilobase of transcript sequence per million reads mapped from library

## Competing interests

The authors declare that we have no competing interests related to this manuscript.

## Authors’ contributions

HCR and LHR cooperated in planning design and analysis strategies for the data presented in this manuscript. HCR generated biological materials used for RNAseq and analyzed the data. HCR and LHR interpreted the data and wrote the manuscript. Both authors read and approved the final manuscript.

## Supplementary Material

Additional file 1 **Supplemental Methods 1.** Preliminary confirmation of hybrid identity of F1 plants via PCR based genetic markers. Click here for file

Additional file 2: Table S3 Genetic positions of reference transcripts showing identity to sequenced markers appearing on a map of *H. annuus* derived from recombinant inbred lines from the population RHA280 × RHA801 (unpublished). Click here for file

Additional file 3 **Supplemental Methods 2.** Perl script used to extract informative single nucleotide variants for analysis of allelic bias in hybrid transcript accumulation. Click here for file

Additional file 4: Figure S1 Venn diagrams showing overlap between full (all data) and reduced (minus outlier samples HA89.9 and F1.TA) analyses. Click here for file

Additional file 5: Table S1 All reference transcripts showing at least one significant difference in analyses of species differences between *H. annuus* cmsHA89 and *H. petiolaris* Pet2152 or analyses of transgressive, non-additive, high variance, or allele-biased transcripts in F1 hybrids. ‘REFERENCE’ identifies the reference contig. ‘LENGTH’ gives the length of the reference contig in bases. Black or grey symbols within the following columns indicate whether the specified difference was statistically significant in both full and reduced analyses (black/bold) or only a single analysis (grey). For “TRANSGRESSIVE” and “NON-ADDITIVE” transcripts, '▲'indicates that F1 samples showed a mean transcript accumulation greater than observed for either parental accession; '▼' indicates lower levels of transcript in F1 samples. For “NON-ADDITIVE” transcripts, '●' indicates that F1 transcript accumulation was intermediate relative to parental accessions. For “ALLELIC BIAS” and “SPECIES DIFFERENCE”, 'A' and 'P' indicate that higher transcript accumulation was observed for the H. annuus or H. petiolaris allele/accession, respectively. For “HIGH CV”, '●' indicates a contig showing a coefficient of variation among F1 samples that is ≥ 2. “TAIR10” provides the best nucleotide BLAST hit to the TAIR10 genome assembly (http://www.arabidopsis.org); “no hit” indicates no results with e-value < e-10. “UNIPROT” provides the uniprot id for the best blastx hit against the UniProt Knowledgebase, release 2012_08. “description” provides an abbreviated annotation of gene function. Click here for file

Additional file 6: Table S2 Transcripts showing transgressive levels of accumulation in F1 hybrids in either full or reduced analyses. Mean RPKM per accession (A = *H. annuus* cmsHA89, H = F1 hybrid, P = *H. petiolaris* Pet2152) are provided for both full (“full”) and reduced (“red”) analyses. ‘SIG’ indicates whether a given transcript shows significant transgression in ‘FULL’, ‘REDUCED’, or ‘BOTH’ analyses, with ‘FULL(TA)’ indicating transcripts that were transgressive in full analyses due to inflation of the F1 mean transcript estimates by the sample F1.TA. ‘Transgressive’ indicates whether F1 transcript levels were determined to be high or low compared to parental accessions. ‘Summary_Annotation’ summarizes the hypothesized gene function based on BLAST-identified similarity. Annotation of the best protein BLAST hit is provided, along with the GenBank identifier and e-value for the BLAST hit, in subsequent columns. Click here for file

Additional file 7: Figure S2 Addendum to Figure 4b, showing full data (outlier samples HA89.9 and F1.TA were not included in the main manuscript figure). This heatmap shows z-score normalized transcript accumulation for 166 reference contigs with CV > 2 within F1 samples. Samples from individual plants are shown in horizontal rows. Hierarchical clustering estimated from Spearman correlation coefficients for pairwise contig (x-axis) and sample (x-axis) distance matrices. The colored bar along the top edge indicates assignment of transcripts to GO Biological Process groups, with prominent categories: green (cell cycle/mitosis), yellow (histones/chromatin modification), blue (metabolism), red (stress/defense), and pink (transcription factors/signaling) Click here for file
