# Supplementary material for: Genome-scale transcriptional analyses of first-generation interspecific sunflower hybrids reveals broad regulatory compatibility
Source: BMC Genomics. 2013 May 23;14:342. doi: 10.1186/1471-2164-14-342 (PMC3679827; doi:10.1186/1471-2164-14-342)
Supplement: Additional file 1 — Supplemental Methods 1. Preliminary confirmation of hybrid identity of F1 plants via PCR based genetic markers. [file 1471-2164-14-342-S1.pdf]

Supplemental Methods

Markers used to verify hybrid parentage of F1 plants were based on length polymorphism of specific amplified fragments of genomic DNA at the loci listed below. DNA was extracted from leaf tissue of 4-6 week old plants using CTAB. DNA was amplified using the locus-specific primers and conditions described below, in 20µl reactions containing 20mM Tris-HCl, 10mM KCl, 10mM $(NH4)_2SO_4$, 2mM MgCl2, 0.1% Triton-X detergent, 2µg BSA (preceeding ingredients supplied as 10X PCR reaction mix), 0.2 mM dNTP mixture, 0.25µM each primer, and approximately 0.1 unit of Taq polymerase. Each reaction contained approximately 20ng of template genomic DNA. Products were visualized on agarose gels as shown in example images below.

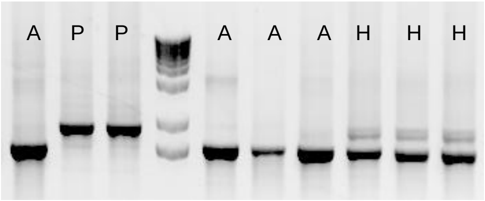

marker name: ETS = external transcribed spacer of the 18s rDNA

reference: Linder et al. 2000 (also Timme et al . 2005)

primers:

ETS1-f 5' CTT TTT GTG CAT AAT GTA TAT ATA GGG GG 3'

18s2l 5' TGA CTA CTG GCA GGA TCA ACC AG 3'

amplification: touchdown protocol from 60-50°C (decreasing 1 degree per cycle over 10 cycles), followed by 30 cycles annealing at 50°C. all cycles are 30s:30s:90s denature (94): anneal: extension (72).

product size: ~ 1650-2000bp (may be more complex, but these are the diagnostic bands). The *H. petiolaris* allele is longer, and tends to look a bit more faint in the heterozygotes (see picture above.) This is shown on a 1% agarose gel run for ~1hr at 180V.

---

marker name: 3724 = unmapped locus from

reference: Strasburg & Rieseberg 2008 Evolution

primers:

3724-F 5' GCCTTCCCGATTATCTTTTC 3'

3724-R 5' TGTTGCATTAGATACAAAAACCA 3'

amplification: 30 cycles x [94°C(30s): 48°C(30s): 72°C(60s)]

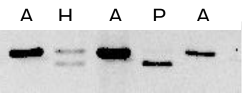

product size: ~ 650bp. the H. annuus allele is longer. We have observed more than one H. petiolaris allele for this marker, but they have all been distinguishably shorter than H. annuus when visualized on a 1.5% agarose gel.
