# Supplementary figures and images for: Genome-scale transcriptional analyses of first-generation interspecific sunflower hybrids reveals broad regulatory compatibility
Source: BMC Genomics. 2013 May 23;14:342. doi: 10.1186/1471-2164-14-342 (PMC3679827; doi:10.1186/1471-2164-14-342)

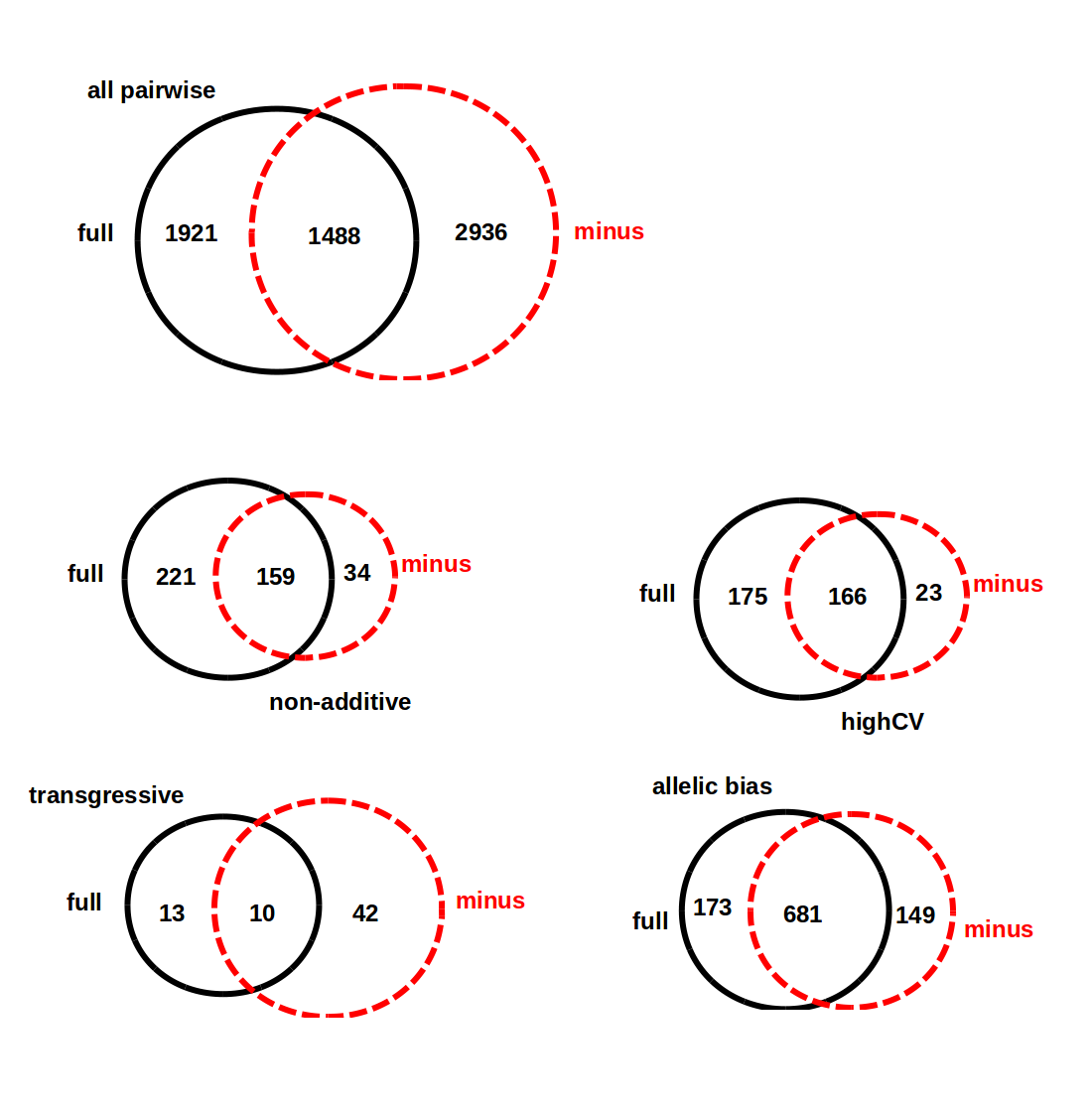

Supplement: Additional file 4: Figure S1 — Venn diagrams showing overlap between full (all data) and reduced (minus outlier samples HA89.9 and F1.TA) analyses. [file 1471-2164-14-342-S4.tiff]

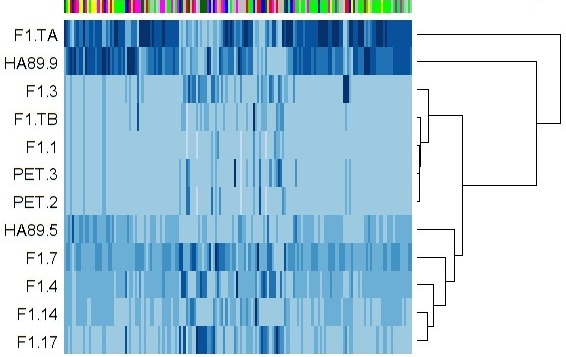

Supplement: Additional file 7: Figure S2 — Addendum to Figure 4b, showing full data (outlier samples HA89.9 and F1.TA were not included in the main manuscript figure). This heatmap shows z-score normalized transcript accumulation for 166 reference contigs with CV > 2 within F1 samples. Samples from individual plants are shown in horizontal rows. Hierarchical clustering estimated from Spearman correlation coefficients for pairwise contig (x-axis) and sample (x-axis) distance matrices. The colored bar along the top edge indicates assignment of transcripts to GO Biological Process groups, with prominent categories: green (cell cycle/mitosis), yellow (histones/chromatin modification), blue (metabolism), red (stress/defense), and pink (transcription factors/signaling) [file 1471-2164-14-342-S7.tiff]
